# A pharmacy too far? Equity and spatial distribution of outcomes in the delivery of subsidized artemisinin-based combination therapies through private drug shops

**DOI:** 10.1186/1472-6963-10-S1-S6

**Published:** 2010-07-02

**Authors:** Justin M Cohen, Oliver Sabot, Kate Sabot, Megumi Gordon, Isaac Gross, David Bishop, Moses Odhiambo, Yahya Ipuge, Lorrayne Ward, Alex Mwita, Catherine Goodman

**Affiliations:** 1 Clinton Health Access Initiative, 383 Dorchester Ave, Boston MA, 02127, USA; 2 Mott MacDonald Group Limited, Croydon, UK; 3 Social Research Division, Steadman Group, Nairobi, Kenya; 4 Tanzania Country Office, Clinton Health Access Initiative, Dar es Salaam, Tanzania; 5 National Malaria Control Program, Ministry of Health and Social Welfare, Dar es Salaam, Tanzania; 6 Kenya Medical Research Institute – Wellcome Trust Research Programme, PO Box 43640, Nairobi, Kenya and Health Policy Unit, London School of Hygiene & Tropical Medicine, Keppel St. London WC1E 7HT, UK

## Abstract

**Background:**

Millions of individuals with malaria-like fevers purchase drugs from private retailers, but artemisinin-based combination therapies (ACTs), the only effective treatment in regions with high levels of resistance to older drugs, are rarely obtained through these outlets due to their relatively high cost. To encourage scale up of ACTs, the Affordable Medicines Facility – malaria is being launched to subsidize their price. The Government of Tanzania and the Clinton Foundation piloted this subsidized distribution model in two Tanzanian districts to examine concerns about whether the intervention will successfully reach poor, rural communities.

**Methods:**

Stocking of ACTs and other antimalarial drugs in all retail shops was observed at baseline and in four subsequent surveys over 15 months. Exit interviews were conducted with antimalarial drug customers during each survey period. All shops and facilities were georeferenced, and variables related to population density and proximity to distribution hubs, roads, and other facilities were calculated. To understand the equity of impact, shops stocking ACTs and consumers buying them were compared to those that did not, according to geographic and socioeconomic variables. Patterning in ACT stocking and sales was evaluated against that of other common antimalarials to identify factors that may have impacted access. Qualitative data were used to assess motivations underlying stocking, distribution, and buying disparities.

**Results:**

Results indicated that although total ACT purchases rose from negligible levels to nearly half of total antimalarial sales over the course of the pilot, considerable geographic variation in stocking and sales persisted and was related to a variety of socio-spatial factors; ACTs were stocked more often in shops located closer to district towns (p<0.01) and major roads (p<0.01) and frequented by individuals of higher socioeconomic status (p<0.01). However, other antimalarial drugs displayed similar patterning, indicating the existence of underlying disparities in access to antimalarial drugs in general in these districts.

**Conclusions:**

As this subsidy model is scaled up across multiple countries, these results confirm the potential for increased ACT usage but suggest that additional efforts to increase access in remote areas will be needed for the scale-up to have equitable impact.

**Trial registration:**

Current Controlled Trials ISRCTN39125414.

## Background

It is estimated that only 37% of patients with malaria seek treatment in the public sector [1], while millions purchase drugs through private outlets. However, artemisinin-based combination therapies (ACTs), the only effective malaria treatment in regions with high levels of resistance to older antimalarial drugs, are very rarely obtained through these private retailers [2]. ACTs are typically sold at retail prices 20-40 times those of common alternatives such as amodiaquine and sulphadoxine-pyrimethamine (SP), making them prohibitively expensive for the 40-60% of febrile individuals seeking treatment from private vendors like pharmacies or drug shops. The widespread development of resistance to cheaper antimalarial drugs means that a majority of individuals suffering from malaria worldwide are not receiving effective treatment.

To encourage scale-up of ACT coverage, the global malaria community is launching the Affordable Medicines Facility – malaria (AMFm) to subsidize the price of ACTs at the point of production for distribution in the public and private sectors. However, concerns remain about whether the intervention will succeed in reaching poor, rural communities [3]. To provide evidence to inform these discussions, the Government of Tanzania and the Clinton Foundation tested the model through a pilot program in two rural Tanzanian districts.

The pilot demonstrated that ACTs could indeed lead to high uptake [4]. Stocking of ACTs in retail drug shops increased from 0/133 in August 2007 to 109/151 (72.2%) a year later. As importantly, these drugs were sold at or below the target retail price; interviewed customers paid an average price of $0.58 during the study period, an amount in line with the cost of other common antimalarial drugs. The effect was striking; while only 1% of antimalarial consumers purchased ACTs before initiation of the subsidy, that fraction increased to about 40% a year later.

Despite these gains, the final survey following 14 months of implementation revealed that 60% of antimalarial drug shop customers still were purchasing alternatives to these heavily subsidised, effective ACTs. This population of individuals receiving ineffective medications can be divided into those who purchased antimalarial or antipyretic drugs at a shop that did not carry ACTs, and second, those who shopped where ACTs were available, but who chose to purchase an antipyretic or another antimalarial instead. By the end of the study period, over a quarter of shops in the study districts continued not to stock ACTs. Of customers buying drugs at shops that did stock ACTs, 44% chose to buy a different antimalarial. With failure rates of upwards of 42% for drugs like amodiaquine [5], it is essential to understand why some shops never stocked ACTs during the subsidy and why some customers did not buy them even when given the option.

This analysis investigates drivers of subsidized ACT stocking and sales at the shop level and ACT purchase at the individual level through spatial analysis. Observed patterning in ACT stocking and sales is compared against that of other common antimalarial drugs to identify factors that may be specifically impacting access to the subsidized drugs. To help contextualize geographic patterns, qualitative data are presented to assess rationales underlying stocking, distribution, and buying disparities.

## Methods

### Study population

The intervention was conducted in two rural districts of Tanzania: Maswa in Shinyanga region and Kongwa in Dodoma region. These districts were comparable in terms of key indicators including population per health facility, employment, prevalence of private drug shops, and bed net ownership. Recent surveys found 30% malaria parasite prevalence in children 6-59 months in Shinyanga, compared to 13% in Dodoma [6]. Socioeconomic status (SES) of households in both districts is below the national average as evidenced by comparison of key assets such as housing materials, toilet facilities, and availability of electricity [7]. Private sector shops – particularly the part II drug stores called *duka la dawa baridi* (DLDB) – provide the majority of fever treatment in Tanzania [8]. DLDB are required to be staffed by an individual with at least one year of health training, and are only allowed to sell over-the-counter (OTC) medicines, although research has shown that both of these requirements are often not met [9]. Drug shops purchase supplies from regional drug wholesalers or pharmacies, which buy from other wholesalers or importers [8,10].

In 2006, Tanzania switched its national guidelines for first-line malaria treatment to ACT, specifically artemether-lumefantrine (AL), with free distribution beginning in the public and non-governmental organization (NGO) sectors in December of that year. ACTs are classified as prescription-only medication, and currently are only consistently available in health facilities and registered part I pharmacies. The previous first-line treatment, SP, was adopted in 2001 but rapidly lost effectiveness [11].

Details of the subsidy design are reported elsewhere [4]. In brief, artemether-lumefantrine, the recommended first-line ACT in Tanzania, was purchased from the manufacturer, Novartis, and sold to a pharmaceutical wholesaler in Dar es Salaam at an average of $0.11 per dose. The wholesaler received no instructions other than to sell the ACTs to drug shops in the two intervention districts according to its standard practices, and it was made clear that the wholesaler would not be monitored or held accountable for its pricing, stocking, or other practices. To reflect potential information, education, and communication interventions that will accompany the AMFm, additional activities included a one-day training of DLDB attendants focused on malaria symptoms and ACT dispensing and dosing, and Population Services International activities emphasizing the importance and availability of ACTs, including local radio advertisements, wall paintings, and themed cultural shows. A suggested retail price of 300, 600, 900, and 1200 Tanzanian Shillings was marked on ACT packages distributed in Kongwa, but not in Maswa.

### Data collection

Retail audits [12] were used to collect data on stocking and sales of antimalarial drugs in all the 226 DLDB that existed in the two districts over the course of the project. DLDB were initially identified through Tanzania Food and Drug Authority records, with unregistered DLDB captured through discussions with local informants and systematic physical reconnaissance throughout each district. All DLDB were georeferenced using hand-held Garmin Etrex global positioning system units.

Each audit involved visiting a DLDB twice at a one month interval. Collectors recorded the stock level of all antimalarial drugs present during each visit, and a short questionnaire was administered to the owner or attendant to determine the amount of each product newly purchased and disposed of (e.g., due to expiry or damage) during the previous four weeks. Sales volumes were then calculated by comparing stock levels between the two visits and adding purchases and subtracting disposals. Data collectors also visited all public and NGO health facilities in each survey period to review ACT stocks and dispensing records. As with DLDB, all locations were georeferenced.

Exit interviews were used to collect information on shoppers and their antimalarial drug choices. Data collectors positioned themselves near a DLDB and remained there for the full business day. Collectors maintained some distance from the DLDB to avoid disrupting normal business. All customers emerging were approached and asked to answer a short questionnaire about the products bought. Those purchasing drugs for malaria or fever were asked about the primary reason they selected the particular antimalarial or antipyretic drug they purchased, and the brand of the product was visually verified. Interviewees were asked a series of questions about 53 types of household assets including ownership of commodities, presence of electricity, and housing materials, in-line with the 2003-04 Tanzania HIV/AIDS Indicator Survey [13]. Both retail audits and exit interviews were conducted a total of four times after the pilot subsidy began in October: November 2007, and March, August, and November 2008.

Finally, qualitative interviews with shop owners and wholesale distributors were conducted in November 2008 following the pilot program to contextualize the quantitative results. Thirty-four DLDB storeowners and four wholesale distributors were interviewed using a semi-structured interview guide. Storeowners were randomly selected from six DLDB groupings constructed according to the number of neighboring shops within 1 km. Distributors were selected by asking the national wholesaler to identify the principal agents it used to supply ACTs in the intervention districts. The structured interviews probed about ACT availability, stocking, pricing, and profitability, and supply of and demand for antimalarial drugs including the subsidized ACT. Interviews were mostly conducted in Kiswahili, transcribed, and then translated into English. Qualitative data were coded and analyzed using MaxQDA; the coding scheme was developed based on the key research questions and themes that were generated by the interviews.

### Variable creation

ArcGIS v9.3 was used to generate spatial variables that could be used to describe the relative remoteness of retail DLDB and their proximity to other DLDB and public facilities. A shapefile of all wards (a “ward” is the 4^th^ administrative boundary in Tanzania) created by the International Livestock Research Institute (ILRI) and National Census Bureau (http://www.ilri.org/gis/) was used to identify the population density for the ward in which each DLDB was located. An additional ILRI shapefile of major roads produced from LANDSAT images was also used.

Euclidean distance from each DLDB to the nearest major road was calculated, as was average distance to the three nearest neighboring DLDB and the number of neighboring DLDB within 1 km. Similarly, the average distance to the three nearest public or NGO facilities and the number within 1 km were computed along with the distance to the nearest facility that was ever found to be stocked with any ACTs and the nearest facility found to be always stocked with at least one dose of ACTs at each survey. The number of surveys in which the nearest facility to each DLDB was stocked was counted. Since DLDB might open or close during the study period, the number of surveys in which each DLDB was found to be open for business was counted as an additional variable that might relate to the health and viability of the shop.

To estimate the real-world distance from each DLDB to the town where the regional wholesaler was located and from which ACT distribution initiated – in Kongwa, this hub was the town of Dodoma, while in Maswa it was Maswa Town – road-weighted distance was calculated as the sum of two figures: shortest-path distance from the hub to the road nearest the DLDB, plus straight-line distance from that road to the DLDB weighted six times as heavily as on-road distance to account for the meandering nature and poorer condition of these roads and therefore the slower speed of these segments of the trip [14]. The weight of 6 was selected based on an assumption that travel by major road could proceed up to the speed limit of 120kph while travel off these roads could achieve a maximum of 20kph; other weights were employed for comparison and did not qualitatively change results (data not shown). Finally, Normalized Difference Vegetation Index (NDVI) values calculated from satellite imagery [15], which represent the amount of visible green vegetation, were given to each DLDB as a measure of the environment in which each shop was located. Log transformations were used to normalize variables that appeared heavily skewed after visual inspection of histograms.

An index of SES was calculated through a principal component analysis of the 53 household asset variables collected in exit interviews [16]. SES was rescaled so that the shopper with the lowest principal component score (and thus lowest status) received a value of 0 and the shopper with the highest score a value of 100. Highest education status attained was considered as a numerical variable ranging from 0 (no formal education) to 6 (university).

### Statistical analysis

All analyses were computed using the SAS System v 9.2.

*Stocking patterns.* DLDB were divided according to whether or not they were ever found to stock or have sold ACTs during any of the four audits following initiation of the subsidy. DLDB that were found to stock or sell ACTs were compared to those that did not in regards to geographic characteristics using t-tests (Satterthwaite tests were used when variances were unequal and pooled tests otherwise) or chi-square tests as appropriate. For example, the average population density at which DLDB stocking or selling ACTs were located was compared to the average for those DLDB not stocking or selling ACTs using a t-test, while the relationship between stocking of ACTs and other antimalarial drugs was compared with a chi-square test. Each of the variables that were found to differ significantly between stocking and not stocking shops in these comparisons then were entered jointly into multivariate logistic regression to determine whether associations were independent.

To examine whether differences in ACT stocking were unique to that subsidized antimalarial drug and its distribution network, these same methods were used to compare stocking of any SP or sulfamethoxypyrazine-pyrimethamine (SMP) product, as well as specifically for the two most commonly sold drug brands besides the subsidized ACT product, the generic drugs Orodar (SP manufactured by Elys Chemical Industries Ltd of Kenya) and Malafin (SMP manufactured by Shelys Pharmaceuticals of Tanzania). Chi-square tests were used to examine whether stocking of ACTs and these other common antimalarial drugs was correlated.

*Buying patterns.* To examine the population of shoppers reached by the subsidized ACT distribution network, the characteristics of individuals shopping at DLDB that stocked and did not stock ACTs were examined through the same statistical methods. Individual-level variables included SES, age, education, and gender of the shopper, the age and gender of the individual for whom the drug was being purchased, and rationale for buying the chosen drug.

Finally, analysis was restricted to the set of DLDB that were found to have stocked or sold ACTs, and the characteristics of individuals who chose to purchase ACTs when they were available at those DLDB were compared to the characteristics of those who purchased other antimalarial drugs. To examine whether different characteristics of individuals were independent predictors of the drug purchased, these variables were entered into a multivariate regression model using the GENMOD procedure in SAS with a REPEATED statement to adjust for the correlation between customers shopping at the same store.

## Results

### Stocking patterns

Significant turnover in the existence of DLDB was observed, with 73 DLDB (32.3%) available for observation in all periods and 52 in only one.

**Figure 1:**
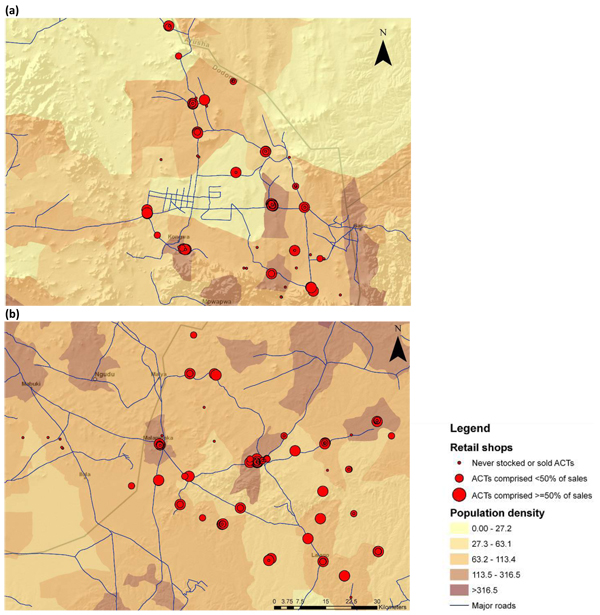
Maps of retail DLDB in (a) Kongwa and (b) Maswa.

*ACT stocking.* Of the 226 DLDB ever surveyed in the two districts, 47 (20.8%) were never found to stock or sell ACTs. 179 (79.2%) were found to stock or sell ACTs during at least one of the four survey periods from November 2007 through November 2008, with the percent stocking increasing steadily over the study period (Table 1). For 93 (52.0%) of these 179 DLDB, ACT sales comprised less than 50% of antimalarial products sold while 86 (38.1%) had ACTs comprise at least 50% of all antimalarial sales (Figure 1). The percentage of DLDB never stocking ACT was higher in Kongwa than in Maswa (28.3% versus 15.7% respectively) (χ^2^=5.25, 1 d.f., p=0.02).

**Table 1:** Characteristics of drug shops and their customers over the four post-subsidy surveys

	November 2007	March 2008	August 2008	November 2008
Shops	138	146	151	166
Newly opened	*	36	32	20
Reopened	*	*	9	18
Closed	*	28	36	23
Stocking ACT	77 (55.8%)	87 (59.6%)	107 (70.9%)	121 (72.9%)
Stocking branded generic SP	110 (79.7%)	96 (65.8%)	116 (76.8%)	129 (77.7%)
Stocking Orodar	85 (61.6%)	62 (42.5%)	82 (54.3%)	88 (53.0%)
Stocking Malafin	30 (21.7%)	26 (17.8%)	52 (34.4%)	65 (39.2%)
Customers surveyed buying drug for malaria or fever	443	415	746	972
Buying antimalarial	292 (65.9%)	290 (69.9%)	455 (61.0%)	572 (58.9%)
Buying ACT	90 (20.3%)	129 (31.1%)	200 (26.8%)	225 (23.2%)
Buying branded generic SP	88 (19.9%)	76 (18.3%)	158 (21.2%)	209 (21.5%)

**Figure 2:**
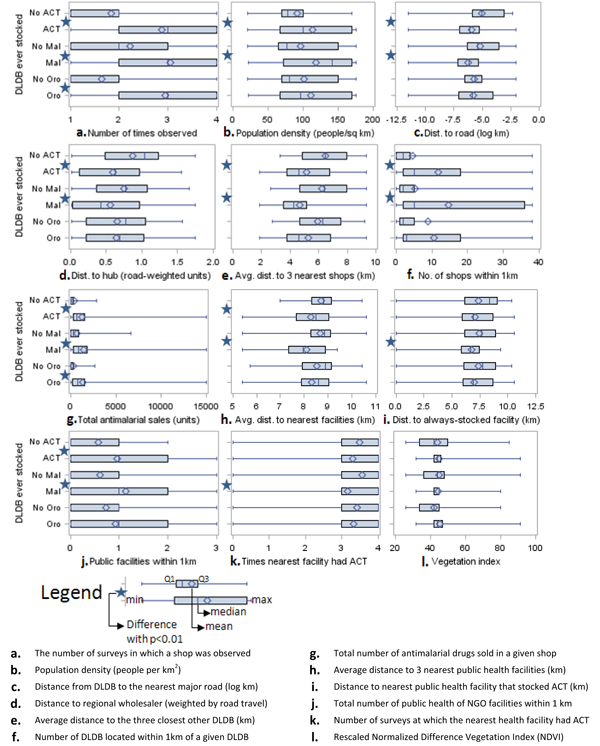
**Distributions of geographic variables according to DLDB stocking status.** The distributions of selected geographic variables are shown for DLDB that were and were not found to stock or sell ACTs, Malafin, and Orodar. Stores stocking and not stocking each product were compared using t-tests, and statistically significant differences (α=0.01) are starred.

On average, DLDB stocking or selling ACTs were less remote than DLDB that never stocked or sold ACTs (Figure 2). For example, the 47 DLDB that were never found to stock or sell ACTs in any of the four surveys were located in wards with average population density of 91.2 people/km^2^, compared to 113.0/km^2^ for DLDB that stocked. Never-stocking DLDB on average were located significantly farther from roads, the district town, and other DLDB and public facilities, and had much lower total antimalarial sales (all p<0.01). DLDB that never stocked were found to be open for business during an average of 1.8 surveys compared to 2.8 for DLDB that ever stocked ACTs (t=-5.93, 224 d.f., p<0.01).

In multivariate logistic regression, three variables, the number of surveys in which the shop was observed (adjusted odds ratio = 1.63, [95% confidence interval = 1.03-2.59]), the distance from the shop to the main town (adj OR = 0.04 [0.01-0.16]), and the average distance to the three nearest neighboring shops (adj OR = 0.61 [0.46-0.81]), remained independent predictors of whether a shop ever stocked ACTs (all p<0.01) with the same direction of association as described above. No other variable in Figure 2 demonstrated a statistically significant association with stocking when controlling for these variables.

*SP stocking.* Only 15/226 DLDB (6.6%) were never found to stock or sell SP or SMP (hereafter referred to as “SP”). Stocking was significantly and positively associated with the number of surveys in which a DLDB was observed, total antimalarial sales, and negatively with the average distance to the three nearest public health facilities (all p<0.01). In multivariate logistic regression, none of the variables depicted in Figure 2 demonstrated statistically significant associations with SP stocking when controlling for the number of surveys in which a shop was observed.

During the four survey periods, retail audits indicated that Orodar and Malafin were purchased 22,003 and 8,016 times, respectively, compared to 37,698 treatments of ACT. There were 49 DLDB (21.7%) that never stocked or sold Orodar, and these DLDB differed significantly from the 177 that did in that they were, on average, surveyed less frequently and had lower total sales (Figure 2). However, no statistically significant patterning in geographic variables was evident (with α=0.01).

There were 106 DLDB that never stocked or sold Malafin (46.9%), with patterns in Malafin stocking and sales extremely similar to those observed for ACTs (Figure 2). The same variables demonstrated significant associations, and in the same direction, as with ACTs with two exceptions: DLDB stocking Malafin were significantly closer to a public health or NGO facility always stocking ACTs, while those stocking ACTs were not, and the public health facility nearest Malafin-stocking DLDB tended to stock ACTs more frequently than was the case for other shops (both p<0.01).

*Joint stocking.* Stocking of ACTs and SP in DLDB was not independent (χ^2^=14.99, 1 d.f., p<0.01). Of the 211 DLDB that stocked SP at any time during the follow-up period, 82.0% also stocked ACTs. In comparison, only 40.0% of the 15 shops not stocking SP had ACTs available. This pattern held true for each of the individual SP products as well; 86.4% of the 177 DLDB stocking Orodar also stocked ACTs compared to 53.0% of those that did not, while 92.5% of the 120 DLDB stocking Malafin also stocked ACTs compared to 64.2% of those that did not. 59.3% of shops stocking Orodar also stocked Malafin, compared to 30.6% of shops that did not (χ^2^=12.70, 1 d.f., p<0.01).

### Consumer characteristics

*Customer characteristics by stocking status of DLDB.* Individuals shopping at DLDB that never stocked or sold ACTs tended to have less education, be of lower SES, and be buying the drugs for slightly older individuals than shoppers at DLDB stocking ACTs (Table 2). The differences in shopper characteristics were consistent when comparing only those shopping in November 2007 with those in November 2008. All three of these variables remained independently associated with stocking status in multivariate regression; a one-level increase in the shopper’s education was associated with adjusted OR = 1.48 (1.18-1.84) for shopping at a DLDB stocking ACTs, while a one-unit increase in SES was associated with adjusted OR = 2.23 (1.65-3.01) and each additional year of the recipient’s age was associated with adjusted OR = 0.97 (0.97-0.99). These relationships were unchanged by additionally controlling for the spatial characteristics of the DLDB locations at which individuals were shopping. No differences in age or education were evident between individuals shopping at DLDB with different stocking or sales of SP; however, as with ACTs, individuals shopping at DLDB never stocking SP had lower SES than those shopping at stores that did.

**Table 2:** Comparison of shopper characteristics according to the stocking status of DLDB at which they shopped

	Never stocked ACTs		Stocked ACTs			Never stocked SP		Stocked SP		
**Variable**	**n**	**Mean (SD)**	**n**	**Mean(SD)**	**t (df), p**	**n**	**Mean(SD)**	**n**	**Mean(SD)**	**t (df), p**

Age of intended recipient (years)	215	23.77(12.95)	1648	21.11(13.85)	2.67 (1861), p=0.01	46	21.37(12.85)	1817	21.42(13.80)	-0.02(1861),p=0.98
Age of purchaser (years)	289	30.43(8.70)	2270	30.11(8.47)	0.60 (2557), p=0.55	53	30.21(7.40)	2506	30.15(8.51)	0.05(2557),p=0.96
Education level of purchaser (0-6)	225	1.81 (0.96)	1966	2.21 (1.06)	-5.38(2189), p<0.01	47	1.94(1.07)	2144	2.17(1.05)	-1.50 (2189),p=0.13
Socioeconomic status of purchaser	289	26.96(11.61)	2274	35.22(16.19)	-10.83^ (444), p<0.01	53	26.63(7.98)	2510	34.45(16.04)	-6.84^(61),p<0.01

^Indicates Satterthwaite t-test was used for unequal variances

*Customer characteristics by drug choice.* Table 3 depicts factors associated with whether or not an individual purchased an antimalarial or an antipyretic and an ACT or a non-ACT at DLDB that stocked them. Individuals buying antimalarials were older, buying drugs for younger recipients, wealthier, and better educated than were individuals buying antipyretics. When entered into multivariate models controlling for correlation between customers at the same store, the customer’s age (adj OR = 1.06 [1.04-1.09]), education (adj OR = 1.32 [1.08-1.61]), and the age of the intended recipient (adj OR = 0.95 [0.93-0.97]) remained independent predictors, but the customer’s SES did not (adj OR = 0.95 [0.79 – 1.14]). ACTs were purchased by older individuals (adj OR = 1.03 [1.01-1.06]) for younger recipients (adj OR = 0.98 [0.96-1.00]) than were other antimalarials, but no differences in sex of the purchaser or recipient were evident (Table 3).

**Table 3:** Characteristics of shoppers purchasing drugs at DLDB that stocked ACTs, by drug choice

	Bought antipyretic		Bought antimalarial			Bought antimalarial other than ACT		Bought ACT		
**Variable**	**n**	**Mean(SD)**	**n**	**Mean (SD)**	**t (df), p**	**n**	**Mean(SD)**	**n**	**Mean (SD)**	**t (df), p**

Age of intended recipient (years)	465	24.00 (12.32)	1002	19.34 (14.27)	6.54^(1036), p<0.01	490	22.35 (13.86)	512	16.46 (14.08)	6.67 (1000), p<0.01
Age of purchaser (years)	720	28.98 (8.71)	1282	30.65 (8.40)	-4.21 (2000), p<0.01	660	30.62 (8.34)	622	30.69 (8.47)	-0.16 (1280), p=0.88
Education level of purchaser (0-6)	629	2.10 (1.02)	1101	2.29 (1.11)	-3.60^ (1404), p<0.01	562	2.29 (1.11)	539	2.29 (1.11)	0.01 (1099), p=0.99
Socioeconomic status of purchaser	720	33.78 (15.01)	1285	37.05 (16.92)	-4.46^ (1642), p<0.01	661	38.44 (17.39)	624	35.57 (16.29)	3.06 (1283), p<0.01

^Indicates Satterthwaite t-test was used for unequal variances

**Figure 3:**
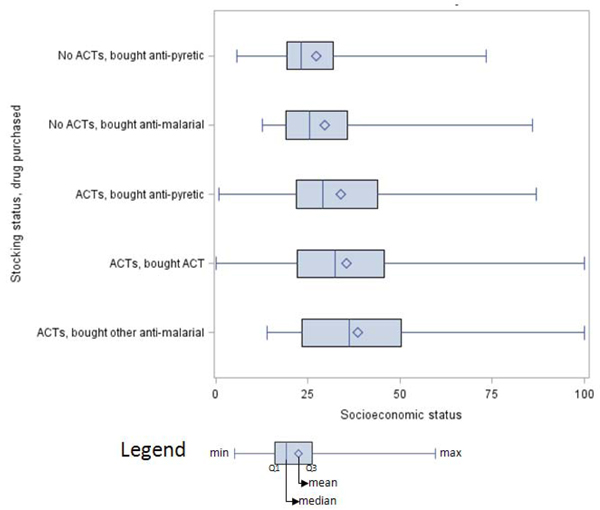
**Distribution of socioeconomic status by stocking status and drug choice.** The distributions of SES are depicted for individuals shopping at DLDB that were stocking and not stocking ACTs and, within those categories, by whether they purchased antipyretics, ACTs, or non-ACT antimalarials.

*Socioeconomic status.* SES varied both according to the stocking status of the store at which the consumer was shopping and according to the type of drug purchased (Figure 3). Individuals buying non-ACT antimalarial drugs at shops stocking ACTs tended to be of significantly higher SES than those buying ACTs; those individuals purchasing ACTs where they were available paid an average of Tsh680 (about $US 0.59) compared to an average of Tsh936 (about $US 0.78) for other antimalarial drugs (t=10.55, 1129 d.f., p<0.01).

*Stocking and shopping rationales.* Of individuals who purchased ACTs at DLDB where they were available and gave a primary reason for doing so, 23.3% (123/529) said the drug was “most effective in treating malaria,” compared to only 15.8% (92/581) who gave that rationale for buying a non-ACT at those same DLDB (χ^2^=9.75, 1 d.f., p=0.002). Individuals who said they selected a drug because of “previous use” were much more likely to purchase a non-ACT (χ^2^=44.09, 1 d.f., p<0.01); 27.2% (158/581) of individuals purchasing non-ACTs gave this rationale compared to 11.3% (60/529) of those buying an ACT. Price was indicated as a reason for buying ACTs for 17.20% (91/529) of customers while only 11.53% (67/581) gave that rationale for purchasing non-ACTs (χ^2^=7.29, 1 d.f., p<0.01). Finally, customers buying ACTs were slightly more likely to have done so because of a recommendation from a seller; 28.0% of ACT buyers (148/529) gave this reason compared to 22.7% of non-ACT buyers (132/581; χ^2^=4.06, 1 d.f., p=0.04).

Of the two interviews conducted at DLDBs never stocking ACTs, rationales for the lack of the subsidized drugs included a perceived lack of demand and a lack of knowledge:

“At first when they were establishing this shop they were not stocking [ACTs], I think there were no customers that is why they hadn’t seen the importance of stocking them”

-*Kongwa DLDB employee*

“I don’t have ACTs, because I have never been educated on them”

-*Maswa DLDB owner*

According to a representative from the wholesaler, drugs were often sold to shopkeepers on credit, so DLDB could not receive more stock until they had paid off what they owed. Therefore, they preferred drugs and products they could sell quickly so they could restock and sell more. Statements citing the importance of the speed of product movement were heard from about half of DLDB shop owners:

“I can’t afford to sell slow moving drugs”

-*Kongwa DLDB owner*

Some perceptions that ACTs were a “slow moving drug” appeared to be related to their relative novelty:

“If customers are used to SP, they will not want ACTs”

-*Maswa DLDB owner*

However, promotions and advertisements on the radio increased awareness of ACTs, and thus were perceived to have increased the desirability for DLDB to stock them in both Kongwa and Maswa:

“The advertisements have really persuaded the public and they will come to the shop and ask for ACTs.”

- *Kongwa DLDB owner*

“Not everyone knows ACT drugs … so if they see the posters or hear it on the radio they can come and buy them.”

-*Maswa DLDB employee*

“People came to know ACTs and so they become a fast moving drug.”

-*Maswa wholesaler*

## Discussion

The results of this investigation both highlight the potential for a subsidy introduced at the top of the private sector supply chain to greatly increase access to effective antimalarial drugs and underscore its reliance upon existing supply chains that currently do not reach all individuals in rural regions. Subsidized drugs were purchased by a majority of customers shopping at stores that stocked them [4] yet disparities in the accessibility of ACTs persisted. As this model is scaled-up to a national level across Africa, it is clear that a deeper understanding of antimalarial drug supply chains and additional interventions that target their behavior will be needed in order to achieve the goal of dramatically increasing ACT access among all those obtaining treatment at private shops.

The two most popular products besides ACTs, although from the same general drug class, manifested widely different stocking patterns. Malafin stocking displayed highly similar geographic patterning to the subsidized ACTs, while stocking of Orodar did not vary by remoteness. There were thus potentially identifiable factors that enabled Orodar to reach rural populations more consistently and equitably than Malafin or subsidized ACTs. Within one year, more shops in the study area stocked the subsidized ACTs than the established products Malafin and Orodar, yet it remains unclear whether subsidized ACTs can rise to the generalized geographic availability of Orodar given more time. Alternatively, the observed differences may be due to fundamentally dissimilar delivery or promotional mechanisms that must be overcome. This understanding is particularly critical for the scale-up of the subsidy since the independent evaluation of the first phase of the AMFm will be of a similarly short time period (less than 18 months of implementation) and thus will be unable to decipher the role of medium- or long-term market factors.

It is possible that different supply chains played an important role in the different patterns observed, though specific characteristics were not captured by this study. Statements by interviewed shopkeepers confirmed that the same wholesaler who distributed the subsidized ACTs was also distributing Malafin, although it is unknown whether Orodar was sold through the same channels. Price was undoubtedly also a driver: Orodar is a significantly cheaper product (exit interviewees paid an average of 778.3 TSh for Orodar compared to 965.9 TSh for Malafin). Counter to local opinion that locally-produced drugs are available more broadly than international ones, Malafin is manufactured in Tanzania while Orodar is produced in Kenya. No data are available on other potential contributing factors including comparative marketing of the products or the amount of time each has been available on the market. Broader analysis of existing supply chains and antimalarial brands should be a priority in preparing for and assessing the scale-up of the subsidy through the AMFm.

Even if subsidized ACTs are able to achieve the reach of Orodar, this analysis suggests that there will be a set of very remote outlets that will be particularly challenging to consistently supply. While shops that never stocked ACTs or SP were a small minority (9/226, 4.0%), they may serve an important role in treating individuals in the most remote areas, distributing an average of 16.2 doses of antimalarials per month. Of 24 individuals buying drugs for fever who were captured by exit interviews, 18 (75.0%) bought antipyretic drugs, and they were poorer than those shopping elsewhere (t=9.01, 26 d.f., p<0.01). The available data indicate two potential causes for the inconsistent supply to these shops. First, they are more remote, more than twice as far, on average, from public health or NGO facilities. Second, they were in operation less, existing in an average of 1.4 surveys compared to 2.7 for other shops (t=3.29, 224 d.f., p<0.01). Both have important implications for potential interventions to reach these shops and their customers; the first suggests the need for supplementation of the inherent market incentives for reaching these areas [17], while the second demonstrates the potential for increased frequency of education [18] and product promotion activities [10].

Several DLDB owners indicating that they did not stock ACTs due to a lack of familiarity with the product. Education programs or advertisements thus could play an important role in improving this component of the supply chain [18]. However, shops in this study were found to open and close with great frequency – only 32% were always surveyed, and nearly a quarter were surveyed only once. Shops that were not observed consistently open for business throughout the year likely were unstable, smaller, and had more volatile stocks. These unstable shops were less likely to stock all of the observed antimalarials, including ACTs. The transitory nature of these shops will make it difficult to provide their owners with the necessary training and education surrounding ACTs, so programs supporting the AMFm may need to consider repeated or periodic programs rather than one-time approaches. Alternatively, it may be more effective to increase access to the formal public sector or engage community health workers in such remote regions [19].

Customers who bought ACTs when given the opportunity to do so were significantly more likely to state that the drugs were most effective, while those who bought non-ACTs were more likely to explain that they preferred to buy familiar medications. These results indicate that education and advertising interventions may prove successful in increasing the probability that customers will buy ACTs when given the opportunity, and ACT purchasing may increase over time without further intervention as individuals become accustomed to the product. The SES of individuals who chose to purchase ACTs when they were stocked was found to be slightly lower than that for individuals choosing to purchase other antimalarial drugs. This difference is attributable to the lower price of the subsidized drugs, which likely made them more accessible. It is important to note that even the less rural individuals reached by this intervention were still quite poor. The majority of customers (72.9%) who succeeded in purchasing ACTs only completed primary school, and these regions are below the national average for key indicators like the percent of households connected to the electricity grid or receiving piped water [7]. It is likely that a similar subsidy may prove even more effective in wealthier, more urban districts.

This analysis has a number of limitations. First, this investigation sought only to examine whether an AMFm-like subsidy would succeed in improving access to ACTs equitably, but it was not designed to evaluate whether such a subsidy constitutes the most appropriate approach; for example, controversy over whether the AMFm will complement the public health system or divert patients from it [20] is not addressed here. This study broadly attempted to capture the most critical variables known to influence drug usage, but it is possible that other important factors were not encompassed. Only DLDB were included in this analysis, so other informal private sector actors were not captured; however, previous studies have indicated that these informal shops are less important sources of anti-malarials in rural Tanzania [21]. The four follow-up surveys of stocking and sales represent only snapshots and as such cannot capture the dynamic nature of patterning. For example, there was enormous turn-over in the DLDB that were found to exist over the course of the year; about a quarter of DLDB only existed during a single survey. Finally, it is possible that the repeated interviews of shop-keepers to assess stocking and sales may have influenced their decision-making, although the absence of ACT uptake in the control district indicates that this bias is unlikely to affect qualitative interpretation of these results. Nevertheless, the patterns observed here provide insight into how a number of varied factors may interact to influence the potential successes and challenges that may occur following the launch of the global ACT subsidy.   

## Conclusions

This research indicates that scale-up of an ACT subsidy to national or global levels has the potential to increase ACT uptake in poor rural areas, but spatial and socioeconomic variation is likely to remain in stocking and sales. Given the launch of the AMFm in 2010, and its potential to effect unprecedented changes in the private sector antimalarial market, it is vital to improve understanding of how these drugs will flow through existing supply chains. In-depth analysis of essential differences in the distribution and use of the most commonly available alternative products such as those discussed here should begin immediately. These results engender cautious optimism that the subsidy will succeed in adding effective drugs into the marketplace at prices in line with older medications, but they emphasize the need to better understand if and how supply chains will need to be augmented or supplemented in order to optimize the impact of the initiative. Performing spatial analyses like those conducted here on an ongoing basis as a component of the monitoring and evaluation of the AMFm may help ensure that inequities in access to treatment are recognized and addressed.

## Trial registration

Current Controlled Trials ISRCTN39125414.

## Competing interests

The authors declare that they have no competing interests.

## Authors’ contributions

JMC conducted the geographic and statistical analyses and drafted the manuscript. OS, MG, YI, LW, AM, and CG conceived of the study and participated in its design and coordination. KS and IG conducted and analyzed the qualitative analyses. DB, MO and LW collected the data. All authors read and approved the final manuscript.
